# Transcripts that associate with the RNA binding protein, DEAD-END (DND1), in embryonic stem (ES) cells

**DOI:** 10.1186/1471-2199-12-37

**Published:** 2011-08-18

**Authors:** Rui Zhu, Michelina Iacovino, Elisabeth Mahen, Michael Kyba, Angabin Matin

**Affiliations:** 1 Department of Genetics, University of Texas, MD Anderson Cancer Center, Houston, TX 77030, USA; 2 Pediatric Hematology-Oncology and Blood and Marrow Transplantation Program, University of Minnesota, Minneapolis, MN 55455, USA

## Abstract

**Background:**

The RNA binding protein, DEAD END (DND1), is essential for maintaining viable germ cells in vertebrates. It is also a testicular germ cell tumor susceptibility factor in mice. DND1 has been shown to interact with the 3'-untranslated region (3'-UTR) of mRNAs such as *P27 *and *LATS2*. Binding of DND1 to the 3'-UTRs of these transcripts blocks the inhibitory function of microRNAs (miRNA) from these transcripts and in this way DND1 helps maintain P27 and LATS2 protein expression. We found that DND1 is also expressed in embryonic stem (ES) cells. Because ES cells share similar gene expression patterns as germ cells, we utilized ES cells to identify additional candidate mRNAs that associate with DND1.

**Results:**

ES cells are readily amenable to genetic modification and easier to culture *in vitro *compared to germ cells. Therefore, for the purpose of our study, we made a genetically modified, stable, human embryonic stem (hES) cell line that expresses hemagluttinin (HA)-tagged DND1 in a doxycycline (dox) regulatable manner. This line expresses modest levels of HA-DND1 and serves as a good system to study DND1 function *in vitro*. We used this stable cell line to identify the transcripts that physically interact with DND1. By performing ribonucleoprotein immunoprecipitation (RIP) followed by RT-PCR, we identified that transcripts encoding pluripotency factors (*OCT4*, *SOX2*, *NANOG*, *LIN28)*, cell cycle regulators (*TP53*, *LATS2*) and apoptotic factors (*BCLX*, *BAX*) are specifically associated with the HA-DND1 ribonucleoprotein complex. Surprisingly, in many cases, bioinformatics analysis of the pulled-down transcripts did not reveal the presence of known DND1 interacting motifs.

**Conclusions:**

Our results indicate that the inducible ES cell line system serves as a suitable *in vitro *system to identify the mRNA targets of DND1. The RIP-RT results hint at the broad spectrum of mRNA targets that interact with DND1 in ES cells. Based on what is known about DND1 function, our results suggest that DND1 may impose another level of translational regulation to modulate expression of critical factors in ES cells.

## Background

Inactivation of the *Dnd1 *gene results in sterility in vertebrates as well as causes development of testicular germ cell tumors in mice [1,2]. *Dnd1 *function is essential to maintain viable germ cells in vertebrates [3,4]. Mice with inactivated *Dnd1 *show progressive reduction in germ cell numbers starting around embryonic day (E) 8 and are therefore rendered sterile at birth. In addition, when inactivation of *Dnd1 *occurs in 129 strain mouse background, these mice have a very high incidence of testicular germ cell tumors [5-7]. Thus, on the 129 background, some germ cells escape death to undergo cancerous transformation. The transformed germ cells eventually differentiate randomly into myriad cell types that constitute the teratomas or teratocarcinomas in the testes. The testicular germ cell tumors in mice resemble human pediatric testicular type I germ cell tumors [8,9].

DND1 has canonical RNA recognition motifs (RRM) [1,10,11]. Mutations engineered in the RRM prevent interaction of DND1 with mRNAs and have also been reported to prevent nucleo-cytoplasmic translocation of zebrafish DND1 [10]. In addition, a disease associated nucleotide polymorphism in the highly conserved RRM of DND1 was detected in a human patient with germ cell tumor [12]. Previous reports have shown that DND1 interacts with the 3'-untranslated region (UTR) of mRNAs such as that of the cell cycle inhibitor, *P27 *(p27^Kip1^, *CDKN1B*) and cell cycle regulator and tumor suppressor, *LATS2 *(large tumor suppressor, homolog 2 of Drosophila - a serine/threonine-protein kinase) [11,13]. DND1 interacts with U-rich sequences found in the 3'-UTR of *P27*. In the case of *P27*, interaction of DND1 hindered miR-221 access to *P27 *3'-UTR. This led to increased expression levels of P27. Although DND1 inhibits mir-372 and 373 from *LATS2 *mRNA, the DND1-binding sequences on the 3'-UTR of *LATS2 *have not been identified. DND1 was also shown to interact with the U-rich sequences on zebrafish *Nanos1 *and *TDRD7 *mRNAs.

The exact mechanism as to how DND1 prevents miRNA-mediated translation repression is unclear. In the case of *P27*, *Nanos1 *and *TDRD7*, the U-rich sequences are found adjacent to miRNA binding sites [10,11]. This suggests that DND1 may bind to mRNA to physically displace the miRNAs and miRISC (miRNA-induced silencing complexes). An alternate possibility is that DND1 may bind mRNA and sequester it away from miRNA access.

In the mouse, DND1 is detected in the early embryo [2,14] and then becomes enriched in primordial germ cells (PGCs) after ~ E8 [3,15]. We also detected DND1 expression in mouse (mES) [14] and human embryonic stem cells (hES). PGCs and ES cells share gene expression patterns, markers and miRNAs in common [16]. For example, both PGCs and ES cells express cellular markers such as OCT4, VASA, NANOG and FRAGILIS. They express similar miRNA families such as miR 209-295, miR 302-367 and miR 17-92 [17-20] and express critical pluripotency factors such as *OCT4*, *SOX2 *and *NANOG *[3,21-24]. Indeed, there is speculation that ES cells may be derived from germ cells of the early embryo [25]. Because ES cells express DND1, it is expected that they also normally express the mRNAs and miRNAs whose activity is modulated by DND1. Moreover, ES cells are easily cultured *in vitro *and are more experimentally amenable compared to germ cells. Therefore, ES cells should serve as a good system to study DND1 function. We therefore sought to determine the mRNA targets of DND1 in hES cells. We expect that there are shared sets of mRNA targets in PGCs and ES cells and knowledge of the nature of the transcripts that associate with DND1 will illuminate the function of DND1 in pluripotent cell types such as in ES and germ cells.

### Methods

#### Generation of stable hES/HA-DND1 cell lines

**Figure 1:**
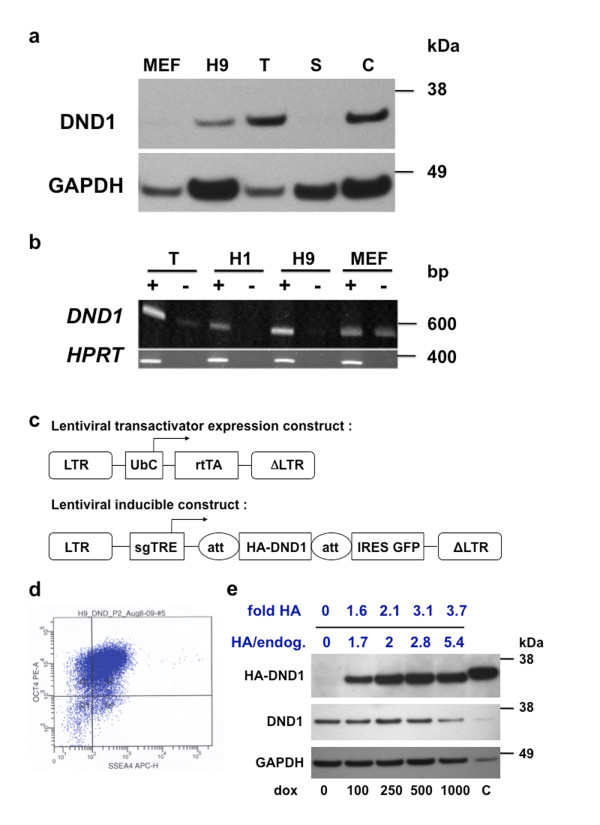
**DND1 expression in hES cells**. (**a**) Immunoblotting using anti-DND1 antibody of MEF, hES line H9, mouse testes (T) and spleen (S) (10 μg protein/lane). Control (C) is 293T cells transfected with HA-DND1 encoding expression vector. (bottom) Blot reprobed with anti-GAPDH. (**b**) RT-PCR to amplify *DND1 *in human testes (T), hES cells H1, H9, and MEFs. (+) lanes had superscript; (-) had no superscript during RT. (bottom) RT-PCR for *HPRT*. (**c**) . The first virus expresses rtTA that binds dox. The second virus carries dox-responsive promoter (sgTRE) that drives HA-DND1 expression. Dox also induces GFP expression, which allows identification of cells carrying both constructs. att (sites for recombinational cloning); LTR and ΔLTR (long terminal repeats); IRES (internal ribosome entry site). (**d**) FACS analysis indicates hES/HA-DND1 cells maintain high expression (77% +ve) of OCT4 and SSEA-4 (quadrant Q2, top right). (**e**) hES/HA-DND1 cells induced to express HA-DND1 upon dox treatment (100 to 1000 ng/mL) for 24 h. Immunoblotting using anti-HA antibody (top panel), anti-DND1 antibody (middle panel) and anti-GAPDH antibody (bottom panel) (20 μg hES/HA-DND1 lysates/lane). Relative HA-DND1 levels (fold HA) range from 1.6 - 3.7 fold with increasing dox. (Relative HA-DND1 of each lane is ratio of HA-DND1/GAPDH). The level of endogenous DND1 (DND1/GAPDH) in hES/HA-DND1 cells treated with 0 to 1000 ng/mL dox was 1.2, 0.9, 1.1, 1.1 and 0.7, respectively. The level of induced HA-DND1 to endogenous DND1 levels (HA/endog) was estimated to be 1.7 to 5.4-fold, with increasing dox.

The hES cell lines H1 (WA01) and H9 (WA09) (WiCell Research Institute, Madison, WI) [26] were cultured on mitotically inactivated mouse embryonic fibroblasts or under feeder free conditions on Matrigel (BD Biosciences). H9 (WA09) hES lines were genetically modified to stably express HA-tagged human DND1 in an inducible manner. Two recombinant lentiviral constructs were introduced into H9 cells. Introduction of the first lentivirus, which expresses transactivator (rtTA) from a constitutive Ubiquitin promoter (UbC), generated H9-rtTA cells [27]. The second virus encodes the inducible target locus (DND1 with HA-tag cloned at 3' end) downstream of the doxycycline-responsive promoter (sgTRE) [28], and bears an IRES-GFP reporter (Figure 1c). The cell line (hES/HA-DND1 cells) containing both viruses was derived by two rounds of transient and low dose doxycycline induction (50 ng/uL, overnight), followed by flow cytometry for GFP+ cells.

Thus, the derived hES/HA-DND1 cells use the tetracycline on (Tet-On) system, in which treatment of cells with doxycycline (dox) causes dox-activated transactivator to interact with the sgTRE element to turn on expression of HA-DND1.

#### FACS analysis

Dual labeling using anti-SSEA-4 and anti-OCT4 antibodies was performed on hES/HA-DND1 cell lines to determine the percent of undifferentiated cells in the population. hES/HA-DND1 cells ranged from 50% to 77% positive (double-labeled) for the markers OCT4 and SSEA-4. These hES/HA-DND1 cells were subsequently passaged and treated with dox prior to performing RIP experiments.

#### Induction of HA-DND1 with increasing concentrations of doxycycline

hES/HA-DND1 cells, grown on Matrigel in 6-well plates, were treated with increasing concentrations, 100 - 500 ng/mL, of doxycycline (dox) for 24 h. The cells were lysed and used for immunoblotting.

*Immunoblotting *with rabbit anti-DND1 antibody, anti-HA antibody and anti-GAPDH was performed as described [14]. Densitometric analysis was performed to quantitate the band intensities.

#### Ribonucleoprotein immunoprecipitation (RIP or RNP immunoprecipitation) assay

**Figure 2:**
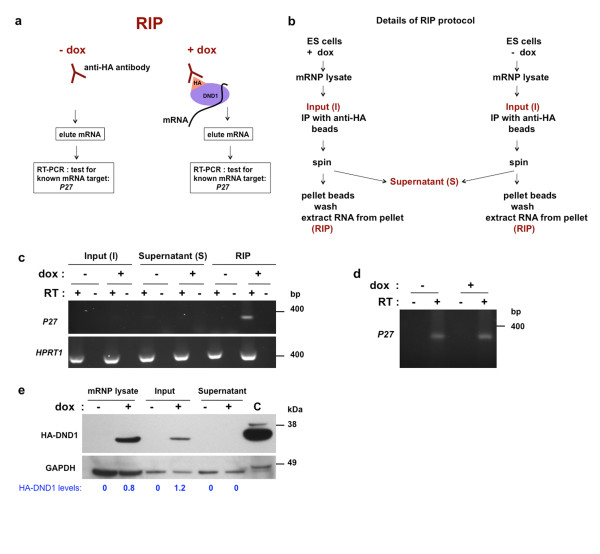
**The RIP-RT procedure**. (**a**) Outline of RIP-RT procedure. hES/HA-DND1 cells were treated with and without dox. Anti-HA antibody was used to pull-down HA-DND1 together with associated mRNA. The mRNA was eluted and RT-PCR used to amplify and detect *P27*. (**b**) Details of the RIP procedure as described in [29]. hES/HA-DND1 cells were used to prepare lysates (mRNP lysates). A fraction of the lysate was used as input (I) for immunoprecipitation (IP) using anti-HA antibody linked to beads. After an overnight incubation, the input was centrifuged. The supernatant (S) was removed. RNA was extracted from the beads (RIP fraction), I and S fractions and used for RT-PCR. (**c**) PCR for *P27 *and *HPRT *on the input (I), supernatant (S) and immunoprecipitation (RIP) fractions. ES cells treated with (+) and without (-) dox were used. Each fraction of the ES cells with and without dox treatment was used to generate cDNA. The RT: + represents presence of Reverse Transcriptase and the RT: - represents no Reverse Transcriptase (control) during cDNA synthesis. **(d) ***P27 *levels in input fraction of untreated (dox-) and treated (dox+) cells when higher PCR cycle numbers (35 cycles) are used. (**e**) (top) Immunoblotting for HA-DND1 in the mRNP lysates, input and supernatant fraction of ES cells treated with (+) or without (-) dox. Control (C) was 293T cells transfected with HA-DND1 encoding expression vector. (bottom) Blots were reprobed with GAPDH. Relative HA-DND1 levels (HA-DND1/GAPDH ratios) in the fractions are indicated at the bottom in blue.

**Figure 3:**
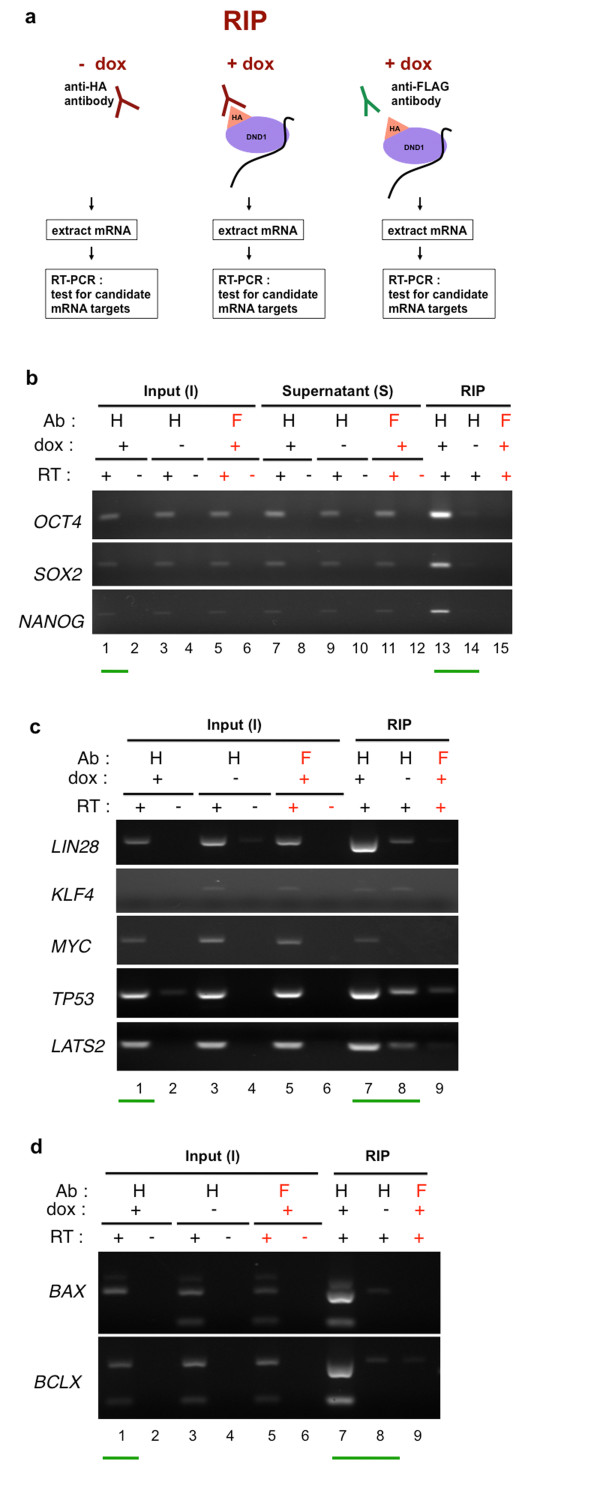
**(a) Outline of RIP-RT and controls**. hES/HA-DND1 cells were treated with dox (+DOX) or not treated with dox (-DOX). The cells were used to make mRNP lysates and the input fraction was incubated with anti-HA antibody or as control, anti-FLAG antibody for RIP. Excess tRNA was added to the input. RNA extracted from the RIP fraction was used for RT-PCR. (**b**) Defining the pluripotency factors which are targets of DND1. Anti-HA antibody (a-H) or anti-FLAG (a-F) was used for RIP. After RIP, RT-PCR was performed on equal amounts of cDNA from I, S and RIP fractions. RT+ indicates presence of Reverse Transcriptase during cDNA preparation. RT- are control lanes with no Reverse Transcriptase. Specific pull down of *OCT4*, *SOX2 *and *NANOG *by anti-HA antibody is observed in lane 13. The PCR cycle number used to detect each transcript is described in Methods. (**c**) Transcripts encoding pluripotency factors and cell cycle regulators targets are targets of DND1. RT-PCR of the S fraction was not performed on these samples. Specific pull down of *LIN28*, *TP53 *and *LATS2 *by anti-HA antibody is observed in lane 7. (**d**) Pro-apototic factor *BAX *and anti-apoptotic factor *BCLX *are specifically pulled down with HA-DND1 during RIP using anti-HA antibodies (lane 7).

RIP was performed essentially as described in [29]. 3 independent RIP experiments followed by RT-PCR were performed and one representative result is shown (Figures 2 and 3). hES/HA-DND1 cells, grown on Matrigel, were treated with dox (500 ng/mL) for 24 h. Control ES cells were untreated. The cells were subsequently lysed and cell extracts were made with mild lysis buffers to minimize exchange of mRNAs between proteins. The lysates (also referred to as mRNP extracts) were incubated with anti-HA antibody (Sigma) linked to agarose beads. Excess tRNA (250 ug yeast tRNA) was included to block spurious interaction of RNAs with HA-DND1. As another control, we incubated mRNP extracts from dox treated cells with anti-FLAG antibody linked to beads (Sigma). The anti-FLAG antibody used was the same IgG1 isotype as the anti-HA antibody.

The beads (linked to anti-HA antibody and now associated with HA-DND1 and RNAs) were pelleted by centrifugation, extensively washed, treated with proteinase K and the RNA was eluted off. The extracted RNA was used to prepare cDNA. RNA concentrations were measured using NanoDrop spectrophotometer (Thermo Scientific) and equivalent amounts were used to prepare cDNA. The 'High capacity RNA-to-cDNA Master Mix with NO RT control' kit (applied biosystems) was used for cDNA preparation. cDNA was also prepared from the other fractions: mRNP lysates, input(I) and supernatant (S) (Figure 2b). Aliquots from these fractions were saved during the RIP procedure.

*PCR *was performed to analyze the RIP, I and S fractions for transcripts encoding *P27*, *HPRT*, etc. The primers for RT-PCR were as follows [30-34]: P27-F ttaaaaatacatatcgctgacttc, P27-R cttcataccccgctccac, OCT4-F gacaacaatgaaaatcttcaggaga, OCT4-R ctggcgccggttacagaacca, SOX2-F cccccctgtggttacctcttc, SOX2-R ttctcccccctccagttcg, LATS2-F gcgacctctgggatgatgtg, LATS2-R ttggagtccccaccagtgaa, NANOG-F atgcctgtgatttgtgggcc, NANOG-R gccagttgtttttctgccac, MYC-F cccgcgacgatgcccctcaac, MYC-R agaagccgctccacatacagtcct, LIN28-F acgtgcgcatggggttcg, LIN28-R tgggccttcagcggacat, KLF4-F aggtggcgggctgatgggcaagtt, KLF4-R gaagcggcagggcagggtgacagt, TP53-F ccccctcctggcccctgtcatctt, TP53-R ccgggcgggggtgtggaatcaac, BCLX-F catggcagcagtaaagcaag, BCLX-R gcattgttcccatagagttcc, BAX-F ggcccaccagctctgagcagatc; BAX-R gacaacccggccccagttgaagtt, HPRT-F gttgagagatcatctccacc, HPRT-R agctatgatgaaccaggtta.

The PCR cycle for each transcript was adjusted depending on the nature of the primer and abundance of the transcript, as determined in preliminary experiments (data not shown). PCR cycles for each transcript was as follows: *P27 *(30 or 35 cycles), *HPRT1 *(35 or 24 cycles), *OCT4, SOX2 *and *NANOG *(26 cycles), *LIN28, KLF4, MYC, TP53, LATS2*, *BAX *and *BCLX *(35 cycles). The expected sizes of the RT-PCR products are: 216 bp for *OCT4*, 232 bp for *SOX2*, 403 bp for *NANOG*, 394 bp for *LIN28*, 424 bp for *MYC*, 347 bp for *KLF4*, 126 bp for *LATS2*, 199 bp for *TP53*, 351 bp xL product from BCLX and 299 bp for *BAX*.

#### DND1 interacting sequences

We examined full-length transcripts for the known human *P27 *DND1 interacting sequence [11] TTTTTCCTTATTT or TTTTTACCTTTT; *Nos1 *DND1 interacting sequence TGTTTGATTTT or TTTTATTTT and *TDRD7 *DND1 interacting sequence TTGATTTT. In cases where multiple transcripts are present, all known transcripts were examined for the presence of any of the known DND1 interacting sequences.

## Results

### DND1 expression in human ES cells

We previously detected DND1 expression in G4 mouse ES cells [14]. Using immunoblotting and RT-PCR techniques, we were also able to detect DND1 expression in human embryonic stem cells (hES), H1 (WA01) and H9 (WA09) (Figure 1a and 1b). However, DND1 is not detected in mouse embryo fibroblast (MEF) cells, the feeder cells on which the hES cells grow. In the case of MEFs, bands are observed in both superscript (-) and superscript (+) lanes (Figure 1b). These likely are non-specific bands and indicate no specific *DND1 *expression in MEFs, as was also previously shown [14].

### Doxycycline-inducible expression of HA-DND1 in hES cells

We established lentiviral modified hES lines that express HA-tagged human DND1 (hES/HA-DND1) in an inducible manner. The hES/HA-DND1 cell line uses the tetracycline on (Tet-On) system in which doxycycline turns on the expression of HA-DND1 [35,36] (Figure 1c). The hES/HA-DND1 cell line showed a modest dose-dependent increase of HA-DND1 levels (1.6 to 3.7 - fold increase of HA-DND1 levels) upon treatment with increasing concentrations of dox for 24 h (Figure 1e, top panel). We also determined the levels of endogenous DND1 expression using anti-DND1 antibody (Figure 1e, middle panel). The level of endogenous DND1 in hES/HA-DND1 cells treated with increasing dox was fairly similar (band intensities were 1.2, 0.9, 1.1, 1.1 and 0.7 with 0 to 1000 ng/mL dox, respectively). Comparing the levels of induced HA-DND1 to endogenous DND1 (HA/endog, Figure 1e), we estimate that HA-DND1 levels range from about a 2 to 6-fold increase compared to endogenous DND1. For subsequent RIP experiments, we treated hES/HA-DND1 cells with 500 ng/mL dox for 24 h which induces a 2.8-fold higher level of HA-DND1 compared to endogenous DND1 levels.

### HA-DND1 associated P27 mRNA

First, we performed RIP [29,37,38] to test whether HA-tagged DND1, induced to be expressed in the hES/HA-DND1 cell line, can pull down *P27 *mRNA. This is because it has been previously shown that DND1 binds to the 3'-UTR of *P27 *mRNA [11].

hES/HA-DND1 cells were treated with dox (500 ng/mL for 24 h) whereas control cells were untreated. The cells were subsequently lysed to make the cell extracts that were used to perform RIP as described in Methods [29] and outlined in Figure 2.

We used RT-PCR to analyze the RIP, I and S fractions for *P27*. cDNA was made using equal amounts (25 ng) of RNA from each fraction (input, supernatant and RIP; +/- dox treated fractions). Equivalent amounts of cDNA were then used for RT-PCR. Our results showed that *P27 *levels were barely detectable in the input fraction (I) but enriched in the RIP fraction (Figure 2c). This indicates that *P27 *mRNA is indeed pulled down specifically with HA-DND1 in the RIP fraction (Figure 2c). Although *P27 *is barely detectable in the input lanes in Figure 2c, P27 is normally present in the hES/HA-DND1 cells as it can be detected in both untreated and dox-treated cells using higher PCR cycles (Figure 2d).

In contrast, RT-PCR for *HPRT *indicated that it is present in all the fractions in dox treated and untreated cells. This rules out specific interaction of *HPRT *mRNA with HA-DND1.

In addition, we monitored for the presence of HA-DND1 in the different fractions. Immunoblotting using anti-DND1 show that the HA-DND1 was present in the dox treated ES cells and in the input (I) fraction, but not in the supernatant (S) fraction (Figure 2e). This indicates that the majority of induced HA-DND1 in the input (I) fraction was efficiently immunoprecipitated by anti-HA antibody and pelleted in the RIP fraction. Because of proteinase K digestion prior to RNA elution, immunoblotting could not be performed on the RIP fraction.

### Pluripotency factors, tumor suppressors and apoptotic factors are DND1 targets

Next, we examined whether transcripts of pluripotency factors associate with DND1. This is because DND1 is normally expressed in both ES cells and primordial germ cells (PGCs) and both of these cell types express pluripotency factors. Thus, we hypothesized that transcripts encoding pluripotency factors may be targets of DND1 in ES cells. Therefore, we performed RT-PCR on RIP fractions to test for pull-down of mRNAs of candidate pluripotency factors such as *OCT4*, *SOX2*, *NANOG*, *LIN28*, *KLF4 *and *CMYC*. We also examined whether the mRNA transcripts of cell cycle regulators and tumor suppressors (*LATS2*, *TP53*) and apoptotic factors (*BAX*, *BCLX*) are targets of DND1.

The results from RIP-RT experiments showed that the following pluripotency factors are specific targets of DND1: *OCT4*, *SOX2, NANOG *and *LIN28 *(Figure 3b and 3c). In the case of *OCT4*, *SOX2, NANOG *(Figure 3b) we observe specific pull down of the transcripts in lane 13 (anti-HA, dox +, RT +) compared to lane 14 (anti-HA, dox -, RT +). In addition we observed enrichment of transcript compared to that in the Input (I) lane (lane 1). In contrast, similar comparison of the transcript levels in the test and control lanes with the input lanes (Figure 3c, lanes 7, 8 and 1), lead us to conclude that *KLF4 *and C *MYC *are not specific targets of DND1. We note that faint bands are observed in the control RIP lanes (Figure 3c, d, lanes 8 and 9). These bands indicate that anti-HA antibody (in -dox cells, lane 8) and anti-FLAG antibody (in +dox cells, lane 9) can also pull down low levels of transcripts, which represent non-specific interactions. In comparison, specific pull-down of transcripts occurs at higher levels (using anti-HA antibody in +dox cells, lane 7).

*TP53 *and *LATS2 *are also specifically pulled down in the RIP fraction of dox-treated cells (Figure 3c, comparing lanes 7 and 8). However, in the case of *TP53 *and *LATS2*, there was not significant enrichment in the RIP fraction (Figure 3c, lane 7) compared to the input (I) lane (Figure 3c, lane 1). This indicates that *LIN28 *is more efficiently pulled down with DND1 compared to *TP53 *and *LATS2*.

On the other hand, transcripts of both the pro-apoptotic factor *BAX *and the anti-apoptotic factor *BCLX *are very efficiently pulled down with DND1 (Figure 3d). There is also significant enrichment of *BAX *and *BCLX *transcripts in the RIP fraction of dox treated cells (Figure 3d, comparing lanes 7 to 8 and 1).

### DND1 binding sequences in the 3'-UTRs of mRNA transcripts

Next, we examined whether the transcripts studied contains the known DND1 binding sequences. We therefore examined the full-length transcripts for U-rich DND1 interacting sequences that were previously mapped in the 3'-UTR of *P27 *[11] as well as for sequences that were mapped to the 3'-UTR of *Nanos1 *and *TDRD7*. In cases where multiple transcripts of a gene are known, all the transcripts were examined for the presence of any of the DND1 interacting sequence. We found that none of the transcripts that we examined in this study contained the same DND1 interacting sequence as *P27*. However, the 3'-UTRs of *OCT4*, *NANOG*, *MYC *and *LIN28 *harbored sequences similar to the DND1 interacting sequences found in *TDRD7*. *LATS2 *3'-UTRs contained the DND1 interacting sequences similar to that found in *Nanos1 *(Table 1).

**Table 1:** Transcripts with potential DND1 binding sequences

Gene	Number of transcripts ^1^	DND1 interacting sequence ^2^	Interaction with DND1 ^3^
***P27 (CDKN1B)***	4	CDKN1B-001 (ENST00000228872) 1196- TTTTTCCTTATTT -1208 1236- TTTTTACCTTTT -1247 CDKN1B-002 (ENST00000442489) 537- TTTTTCCTTATTT -549	**+**

***HPRT1***	3	**-**	**-**

***OCT4 (POU5F1)***	6	POU5F1-001 (ENST00000434616) 1345- TTGATTTT -1352 POU5F1-002 (ENST00000412166) 1088- TTGATTTT -1095 POU5F1-004 (ENST00000463773) 621- TTGATTTT -628	**+**

***SOX2***	1	**-**	**+**

***NANOG***	3	NANOG-001 (ENST00000229307) 2042- TTGATTTT -2049	**+**

***LIN28***	2	LIN28A-001 (ENST00000326279) 3879- TTGATTTT -3886 LIN28A-201 (ENST00000254231) 3326- TTGATTTT -3333	**+**

***KLF4***	7	**-**	**-**

***MYC***	5	MYC-001 (ENST00000377970) 2283-TTGATTTT -2290	**-**

***TP53***	15	**-**	**+**

***LATS2***	3	LATS2-001 (ENST00000382592) 4176- TTTTATTTT -4184	**+**

***BAX***	12	**-**	**+**

***BCLX (BCL2L1)***	10	**-**	**+**

^**1 **^Number of expressed transcripts as reported in Ensembl (http://www.ensembl.org/Homo_sapiens/Info/Index), not including duplications

**^2 ^**The specific transcript(s) that encodes previously described DND1 interacting sequences [11]. The nucleotide position of the DND1 interacting sequence within the full-length transcript is indicated.

^3 ^As experimentally determined and shown in Figure 2 and 3.

Thus, sequence examination of transcripts alone does not predict whether a particular transcript will bind to and be pulled-down with DND1. Although *MYC *did not bind to HA-DND1, the 3'-UTR of one of its transcripts contains putative DND1 binding sequences. In contrast, even though *BAX *and *BCLX *bound very efficiently to DND1 ribonucleoprotein complex, we did not find DND1 binding sites in any of its transcripts. This suggests that there likely exists additional DND1 binding sequences that are yet to be defined. To test this, we aligned the 3'-UTRs of the DND1 interacting mRNAs to examine for any common sequences. We found some regions with weak consensus that may represent novel DND1 binding sites. Further experimental analysis will be required to establish whether these are indeed DND1 binding sites.

The other possibility is that interaction of mRNAs with DND1 may be mediated through co-interaction of DND1 with other proteins and thus presence of DND1 binding sequences within mRNAs may not be essential for interaction.

## Discussion

We demonstrate use of a stable, inducible ES cell line to identify transcripts that associate with DND1. Our results show that transcripts of pluripotency factors associate with DND1. OCT4, SOX2 and NANOG are key transcription factors essential for maintenance of the pluripotent state of ES cells [39-41] and are also important for early germ cells [3,22,24]. These results lead us to hypothesize that DND1 likely imposes another level of regulation of expression of pluripotency factors. However, it remains to be determined how DND1 association with these transcripts affect ES cell properties.

miRNAs target multiple mRNAs simultaneously. On the other hand, DND1 is also able to inhibit miRNA activity from multiple mRNAs [11,13]. Our results also hint at the broad range of mRNA targets of DND1. Further studies are needed to elucidate the role of DND1 in modulating specific miRNA activity from each mRNA.

Our RIP-RT results show that the transcripts of the cell cycle regulators, *TP53 *and *LATS2 *mRNA are also targets of DND1. This observation is corroborated by a recent study where RIP technique was applied to NIH3T3 cells and showed that tagged DND1 is able to pull down a number of cell cycle regulators including *Lats2 *and *p53 *[42].

In addition, mRNA transcripts of both the pro-apoptotic factor *BAX *and the anti-apoptotic factor mRNA, *BCLX*, are efficiently pulled down with DND1. Thus DND1 interacts with a broad variety of targets and with mRNAs that have opposite effects, as for example, DND1 associates with mRNAs of both anti- and pro-apoptotic factor, *BCLX *and *BAX*, respectively. This raises the question as to what confers specificity for DND1 function? One possibility is that there exist factors that favor interaction with specific mRNAs at particular stages of the cell cycle or under certain physiological conditions. It is also likely that intrinsic and cellular factors may regulate DND1 function to promote cell survival and maintain a pluripotent cell state. Depending on the physiological state of the cell, the balance of expression of the opposing factors likely determine cell fate, death or transformation.

Germ cells, testicular cancer cells and ES cells share gene expression patterns [21,24,43]. For example, *SOX2*, *NANOG *and *LIN28 *expression can be used to distinguish different histological subtypes of human testicular cancers [44,45]. This is thought to reflect the origin of testicular cancer from germ cells. Whether the same pluripotency factors also associate with DND1 in germ cells will have to be experimentally determined. Interestingly, our observation that DND1 interacts with pluripotency and anti-apoptotic factor mRNAs may explain why lack of DND1 results in the death of germ cells in mice. Pluripotency factors are able to reprogram differentiated adult cell types into iPS (induced pluripotent stem) cells. For example, human iPS cells require *OCT4, SOX2, NANOG*, and *LIN28 *whereas mouse iPS cells require *Oct4, Sox2, Myc *and *Klf4 *[46,47]. Interestingly, we found that transcripts encoding *OCT4, SOX2, NANOG *and *LIN28 *but not *KLF4 *associate with DND1 in hES cells.

Surprisingly, we found that DND1 interacting sequence similar to *P27 *are rarely present in other transcripts. However, the 3'-UTR of some transcripts harboured DND1 interacting sequences described in *Nanos1 *and *TDRD7*. Therefore, at present, based on sequence analysis alone, it is difficult to predict whether a particular transcript interacts with DND1.

## Conclusion

In conclusion, we report the generation of the inducible hES cell system (hES/HA-DND1 cells) and demonstrate the advantage of using this stable, dox inducible hES cell system to identify the RNA targets of DND1. hES cells closely resemble primordial germ cells and we can induce and control expression of modest levels of HA-tagged DND1. Using the hES/HA-DND1 cells, we demonstrate that transcripts of pluripotency factors, cell cycle regulators and apoptotic factors are associated with DND1 and are likely targets of regulation by DND1. We found that RIP followed by RT-PCR allows us to quickly and readily discriminate between specific and non-specific mRNA targets of DND1. Further techniques such as microarray or RNA-Seq following RIP will be useful to identify the global targets of DND1 from ES cells. Although a number of transcripts associate with DND1, surprisingly, DND1 interacting sequence similar to *P27 *are rarely present in other transcripts. Thus additional DND1 interacting sequences remain to be identified.

Our result suggests that DND1 imposes another level of translational regulation that may modulate expression of critical factors in ES cells. Because DND1 is implicated in reciprocally regulating miRNA mediated translation suppression of specific target mRNAs, this suggests that the balance between DND1 and miRNAs modulates context-dependent mRNA targets.

## Abbreviations

DND1: (dead end homolog 1 (zebrafish)); HA-DND1: (hemagluttinin-tagged DND1); *P27 *(*CDKN1B*: cyclin-dependent kinase inhibitor 1B, Kip1); *LATS2*: (large tumor suppressor, homolog 2 of Drosophila); *OCT4 *(*POU5F1*: POU class 5 homeobox 1); *SOX2*: (SRY (sex determining region Y)-box 2); *NANOG: (*Nanog homeobox); *LIN28*: (lin-28 homolog A (C. elegans)); *KLF4*: (Kruppel-like factor 4); *CMYC*: (v-myc myelocytomatosis viral oncogene homolog (avian)); *TP53*: (tumor protein p53); *BCLX *(*BCL2L1*: BCL2-like 1); *BAX*: (BCL2-associated × protein); *HPRT*: (hypoxanthine phosphoribosyltransferase 1); GAPDH: (glyceraldehyde-3-phosphate dehydrogenase); dox: (doxycycline); RIP: (ribonucleoprotein immunoprecipitation).

## Authors' contributions

RZ characterized the hES/HA-DND1 cells, performed the RIP experiments and participated in writing of the manuscript. MK conceived the design and generation of hES/HA-DND1 cells. MI, EM and MK generated the hES/HA-DND1 cells.

AM conceived the study, participated in its design and coordination and wrote the manuscript. All authors read and approved the final manuscript.
